# A Single-Institution Experience in the Use of Chest Radiographs for Hospitalized Children Labeled as Asthma Exacerbation

**DOI:** 10.3389/fped.2021.722480

**Published:** 2021-08-19

**Authors:** Ela Beyyumi, Mohamed I. Tawil, Huda AlDhanhani, Sara Jameel, Manal Mouhssine, Hasa M. AlNuaimi, Osama Hamdoun, Amnah Alabdouli, Mohammed T. Alsamri, Ghassan A. Ghatasheh, Taoufik Zoubeidi, Abdul-Kader Souid

**Affiliations:** ^1^Department of Pediatrics, Tawam Hospital, Al Ain, United Arab Emirates; ^2^Department of Radiology, Sheikh Khalifa Medical City, Abu Dhabi, United Arab Emirates; ^3^Department of Statistics, College of Business and Economics, UAE University, Al Ain, United Arab Emirates; ^4^Department of Pediatrics, College of Medicine and Health Sciences, UAE University, Al Ain, United Arab Emirates

**Keywords:** asthma, asthma exacerbation, chest radiograph, radiation, quality improvement, respiratory infection, diagnostic radiation, imaging

## Abstract

**Background:** Risks of diagnostic radiation have become more notable lately, particularly in young children with chronic medical conditions. This study reports on the cumulative radiation from chest radiographs in children with asthma. Its main purpose was to review our current practice and suggest minimizing the use of chest radiographs.

**Methods:** The study was retrospective and conducted at a pediatric tertiary center. Eligibility criteria included children 2–15 y, admitted between January 2017 and December 2018 for asthma management.

**Results:** Of the 643 children admitted as “asthma exacerbation,” 243 [40% females; age (mean ± SD) 5.4±3.3 y] met the study criteria for inclusion. Ninety-two (38%) children had a temperature of 38.8±0.7°C on the day of admission. Antibiotics were prescribed for 148 (61%) children, mainly for presumed pneumonia. Chest radiographs were requested for 214 (88%) children, mainly on the day of admission. Only 38 (18%) chest radiographs showed focal/multifocal pneumonia justifying antibiotic use. Significant predictors for requesting chest radiographs were antibiotic use for presumed pneumonia, lower oxygen saturation at presentation, and a requested blood culture. The rate of chest radiographs per year was negatively related to the child's age; the younger the child the higher the rate (model coefficient −0.259, *P* < 0.001). For children < 5 y, the rate of chest radiographs was 1.39 ± 1.21/y and radiation dose 0.028 ± 0.025 mSv/y. The corresponding rates for children ≥5 y were 0.78 ± 0.72/y and 0.008 ± 0.007 mSv/y, respectively (*P* < 0.001).

**Conclusion:** Chest radiographs were commonly requested for children with asthma, especially younger children. Prospective studies are necessary to measure the impact of this practice on the children's health.

## Introduction

Compared to adults, young children are more sensitive to the damaging effects of low-level ionizing radiation produced by medical imaging (1). Although the negative impact of such repeated exposures is yet to be measured systematically (2), the consensus is that diagnostic radiographs should be kept at a minimum, especially for young children with a chronic condition such as asthma^1^. Childhood asthma is a leading cause of hospitalization and disparities in its management are substantial (3).

The American Academy of Pediatrics (AAP), the National Heart, Lung and Blood Institute (NHLBI), and the Global Initiative for Asthma (GINA) have issued statements addressing hospital-based care for children with asthma (4, 5)^2^. The consensus is, “all radiographic imaging must be justified” (https://www.icrp.org/docs/ICRP_Publication_103-Annals_of_the_ICRP_37(2-4)-Free_extract.pdf). There is an urgent need to improve the awareness of radiation dosing and communicating its risk with children's families (6, 7).

Respiratory disorders are especially common in the UAE (United Arab Emirates) (8). In one study, the estimated prevalence of childhood asthma was about 13% (9). Many of these children, however, have joint causes, such as atopy, infection, and inherited entities such as primary ciliary dyskinesia (10). Nevertheless, the presumptive diagnosis of ‘asthma exacerbation' remains a primary cause of our pediatric admissions (11). This study aims to determine the necessity of requesting chest radiographs for these children. Its main objectives are to explore our current practice and suggest rules for improvement.

## Methods

This retrospective data collection study was conducted at Tawam Hospital, a tertiary referral center in Al Ain (Abu Dhabi, UAE) that covers a population of over 600,000. The study was approved by “Tawam Human Research Ethics Committee” (AA/AJ/784; “antibiotics and radiographic investigation misuse in children admitted with acute asthma exacerbations, Tawam Hospital experience”). Informed consent to participate in this “Retrospective Chart Review” was exempt. Our research question (hypothesis) was the necessity of chest radiograph for children admitted with asthma exacerbation.

In our institution, the equipment was expected to result in a radiation dose of 0.02 mSv per chest radiograph for children <5 y, and 0.01 mSv per chest radiograph for children ≥5 y. For the purpose of this study, all abnormal chest radiographs were independently reviewed by the pediatric radiologist (M.I.T.); the findings are summarized in Results.

The participants were children 2 to 15 y who were admitted to the pediatric ward between January 2017 and December 2018. They had a primary diagnostic label of “asthma exacerbation” and received corticosteroids as a treatment for asthma. Only the last admission (most recent and thus most likely to capture the current practice) was considered for those who had multiple hospitalizations during the study period. Children with group A streptococcus pharyngitis (*n* = 9) or positive mycoplasma IgM antibody (*n* = 3) were included, as these pathogens could trigger asthma. Children who were admitted to the pediatric intensive care unit (n = 35) or did not receive corticosteroids (*n* = 155) were excluded. Other exclusion criteria were children with Down syndrome, complex heart disease, swallowing dysfunction, sickle cell disease, primary ciliary dyskinesia, interstitial lung disease, cystic fibrosis, Stuve-Wiedemann syndrome, chronic lung disease of prematurity, tracheostomy, bronchiectasis, pulmonary hypertension, malignancy, tracheoesophageal fistula, and diaphragmatic hernia.

Nasopharyngeal swabs were performed on admission for viral antigen detection [typically included influenza A and B, respiratory syncytial virus (RSV), parainfluenza, and adenovirus] and/or for pathogen genome detection by real-time one-step polymerase chain reaction (RT-PCR). The latter methodology used Allplex™ Respiratory Panels One and Two (Seegene Biotechnology Inc., Seoul, Korea). Panel One included influenza A, influenza B, human RSV A and B, influenza A subtypes H1, and H1pdm09. Panel Two included adenovirus, metapneumovirus, enterovirus, and parainfluenza viruses 1, 2, 3, and 4.

## Statistics

The analysis was performed using SPSS (version 20). Multiple logistic regression of requesting chest radiographs vs. various predictors was performed using backward selection (likelihood ratio). Similarly, multiple logistic regression of using antibiotics vs. various predictors was performed using backward selection (likelihood ratio). Analysis of the rate of chest radiographs per year was performed using a negative binomial model with log(age) as offset variable (sample size = 243). The explanatory variables were age and age^2^. *P* < 0.05 was considered significant.

## Results

A total of 643 children were admitted with the label “asthma exacerbation” during the study period. Of those, 243 children met the study eligibility criteria and their characteristics are summarized in Table 1. Three blood cultures were positive; one for *viridans streptococci* (a 4-year-old child with a temperature of 39.2°C), one for micrococcus (a 7-year-old child who was afebrile), and one for unspecified “gram positive rods” (a 4-year-old child with temperature 38.0°C). Antibiotics were prescribed for 148 (61%) children, mainly for presumed pneumonia (48%); three of these children had positive mycoplasma IgM antibody. Five (2%) children received antibiotics for otitis media, nine (4%) for group A streptococcus pharyngitis, and fourteen (6%) as an empiric dose of ceftriaxone or amoxicillin/clavulanic acid in the emergency department. The duration of antibiotics was 8.4 ± 2.8 days (median, 10 days). The most commonly used antibiotic was amoxicillin/clavulanic acid, followed by ceftriaxone, cefuroxime, amoxicillin, penicillin V, azithromycin, and clarithromycin.

**Table 1:** Study sample population (*n* = 243).

**Age (years)**	
Mean ± SD	5.4 ± 3.3
Median	4.0
Range	2.0 to 15.0
Females	98 (40%)
Fever (≥38.0^°^C) on the day of admission	92 (38%)
Oxygen saturation ≤ 92% on the day of admission	59 (24%)
Chest radiograph request during this hospitalization	214 (88%)
Chest radiograph request during this hospitalization for children <5 years of age	113 (91%)
Chest radiograph request during this hospitalization for children ≥5 years of age	101 (85%)
**Number of views**	
One	196 (92%)
Two	18 (8%)
Abnormal chest radiograph results (please see Results)^d^	97 (46%)
**Number of chest radiographs per year for children <5 years of age**	
Mean ± SD	1.39 ± 1.21
Median	0.97
Range	0.22 to 6.00
**Number of chest radiographs per year for children ≥5 years of age**	
Mean ± SD	0.78 ± 0.72
Median	0.50
Range	0.00 to 3.64
**Radiation exposure (mSv per year) for children <5 years of age^e^**	
Mean ± SD	0.028 ± 0.024
Median	0.020
Range	0.004 to 0.120
**Radiation exposure (mSv per year) for children ≥5 years of age^e^**	
Mean ± SD	0.008 ± 0.007
Median	0.005
Range	0.000 to 0.036
Blood culture request during this hospitalization^a^	173 (71%)
Antibiotic administration during this hospitalization	148 (61%)
**Clinical reasoning for the antibiotic use (*n* = 149)**	
Presumed pneumonia^b^	115 (48%)
Acute otitis media	5 (2%)
Group A streptococcus pharyngitis	9 (4%)
An empiric dose in the emergency department	14 (6%)
Others^c^	5 (2%)
**Duration of the antibiotic use (days)**	
Mean ± SD	8.4 ± 2.8
Median	10.0
Range	1.0 to 16.0
**Nasopharyngeal swabs for viral antigen detection**	
Positive^f^	22 (14%)
Negative	131 (86%)
Not done	90 (37%)
**Nasopharyngeal swabs for pathogen genome detection by PCR**	
Positive^g^	18 (7%)
Negative	9 (4%)
Not done	216 (89%)
**Admission length of stay (days)**	
Mean ± SD	2.7 ± 1.74
Median	2.0
Magnesium sulfate use during this hospitalization	18 (7%)
Previous pediatric ward admission within 1 month	18 (7%)
Previous ICU admission	25 (10%)

a *Three blood cultures were positive: Viridans streptococci, micrococcus, and unspecified “gram positive rods”; all received antibiotics for a presumed pneumonia*.

b *Three children had positive mycoplasma IgM antibody*.

c *Acute sinusitis, acute bronchitis, and lymphadenitis*.

d *Based on the chest radiograph report, “normal” vs. “abnormal.” Please see Results for further details*.

e *Using a radiation dose of 0.02 mSv (Sievert = 1 J.kg^−1^) per chest radiograph for children <5 years of age and 0.01 mSv per chest radiograph for children ≥5 years of age*.

f *These included influenza A (n = 6), influenza B (n = 4), both (n =2), respiratory syncytial virus (RSV, n = 6), parainfluenza (n = 3), and adenovirus (n = 1)*.

g *These included RSV (n = 3), adenovirus (n = 2), influenza A (n = 2), enterovirus (n = 2), rhinovirus (n = 1), RSV plus streptococcal pneumonia (n = 1), RSV plus haemophilus influenzae (n = 2), RSV plus enterovirus plus influenza A plus parainfluenza (n =1), metapneumovirus plus haemophilus influenzae (n = 2), rhinovirus plus streptococcal pneumonia (n = 1), adenovirus plus haemophilus influenzae bocavirus (n = 1)*.

Pathogen studies were requested for one hundred and eighty (74%) children; forty (16%) children had a positive test (Table 1). Temperature (36 to 41°C) on the day of admission did not correlate with either the white blood cell count or the C-reactive protein (*R*^2^ < 0.03 for both).

Chest radiographs were requested for 214 (88%) children. Eighteen (8%) children had two views. One hundred seventeen (54%) chest radiographs were normal. Ninety seven (46%) chest radiographs were interpreted as abnormal and were reviewed by the pediatric radiologist. His evaluations were as follows; “frontal chest radiographs of 97 children were available for review. Previous chest radiograph(s) were available for comparison in 95 children. The radiographic findings were classified into three categories: (1) Normal, when no radiographic abnormalities were identified; (2) Pneumonia, when either unifocal or multifocal consolidation was identified; and (3) Small airways disease related changes, when the chest radiograph showed one or more of the following radiographic features: (a) Hyperinflation, (b) Perihilar bronchial wall thickening, or (c) Long standing bands of atelectasis. The results show seven chest radiographs were interpreted as normal, 52 as small airways disease related changes, and 38 as pneumonia (of which 27 as focal pneumonia and 11 as multifocal pneumonia).”

The adjusted rate of chest radiographs for children <5 y was 1.39 ± 1.21/y and ≥5 y was 0.78 ± 0.72/y (*P* = 0.000). The corresponding radiation dose was 0.028 ± 0.025 mSv/y and 0.008 ± 0.007 mSv/y, respectively (*P* = 0.000). The cumulative radiation was five-fold higher in children <5 y than that in children ≥5 y (*P* = 0.000).

Significant predictors for requesting chest radiograph were use of antibiotics for presumed pneumonia (*P* = 0.002), lower oxygen saturation at presentation (*P* = 0.031), and requesting blood culture (*P* = 0.035), Table 2. The significant predictor for using antibiotics was abnormal chest radiograph (*P* = 0.009), Table 3.

**Table 2:** Multiple logistic regression of requesting chest radiograph (“yes”) vs. predictors listed in the footnote, using backward selection (likelihood ratio); *P*-value for the model is <0.001.

	**B**	**S.E**.	**Wald**	**df**	***P***	**Exp(B)**	**95% C.I. for Exp(B)**	
							**Lower**	**Upper**
Reason of antibiotic			11.667	3	0.009			
Presumed pneumonia	2.069	0.657	9.913	1	0.002	7.916	2.184	28.695
Acute otitis media	−1.090	1.281	0.725	1	0.395	0.336	0.027	4.137
Other reasons	0.038	0.739	0.003	1	0.959	1.039	0.244	4.426
Oxygen saturation at presentation	−0.154	0.071	4.647	1	0.031	0.857	0.745	0.986
Requesting blood culture	1.068	0.507	4.440	1	0.035	2.909	1.077	7.855
Requesting viral antigen test	0.937	0.508	3.404	1	0.065	2.553	0.943	6.908

*Variables entered on step 1: Age, gender, oxygen saturation at presentation, antibiotic use, reason for the use of antibiotics, temperature at presentation, requesting viral studies, requesting blood culture, length of hospital stay (days)*.

**Table 3:** Multiple logistic regression of using antibiotics (“yes”) vs. predictors listed in the footnote, using backward selection (likelihood ratio); *P*-value for the model is 0.001.

	**B**	**S.E**.	**Wald**	**df**	***P***	**Exp(B)**	**95% C.I. for Exp(B)**	
							**Lower**	**Upper**
Chest radiograph result (normal)	−1.542	0.591	6.802	1	0.009	0.214	0.067	0.682
Length of hospital stay (days)	0.350	0.215	2.653	1	0.103	1.418	0.931	2.160

*Variables entered on step 1: Age, gender, oxygen saturation at presentation, magnesium sulfate use, temperature at presentation), leukocyte count, CRP (mg/L), number of chest radiograph views, chest radiograph result, requesting viral studies, results of viral studies, requesting blood culture, length of hospital stay (days), previous ICU admission(s), admission within 1 month, and number of admissions over the past 12 months prior to encounter*.

**Figure 1:**
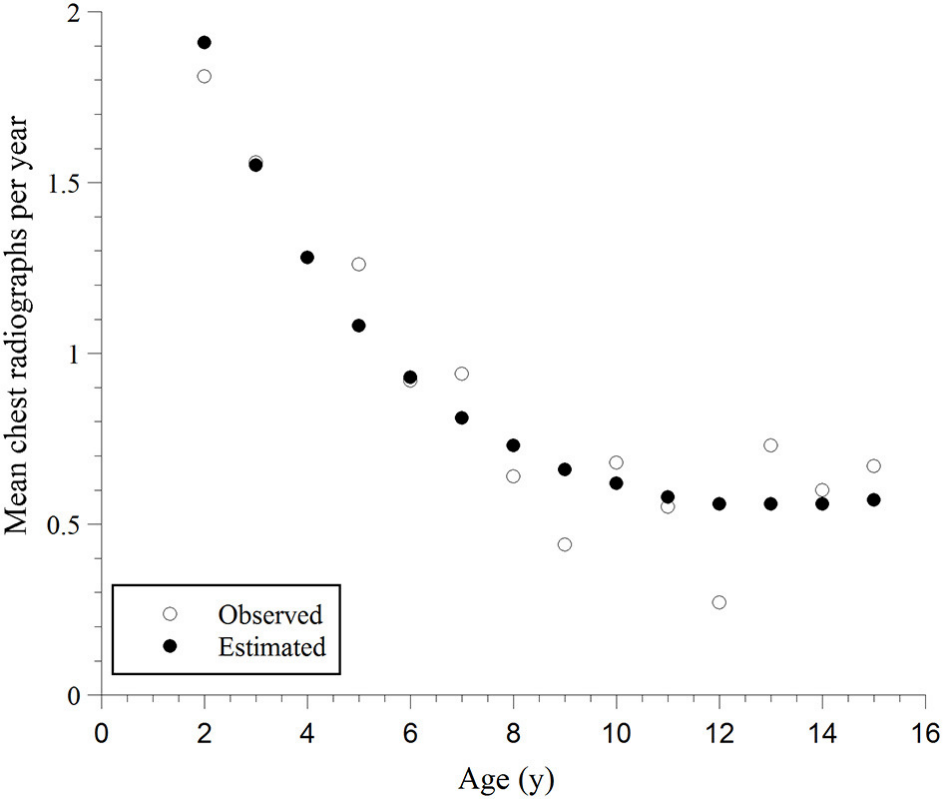
Mean observed rates of chest radiographs per year and the corresponding predicted rates, estimated using the negative binomial regression model vs. age and age^2^; the model fitting curve is: Rate of chest radiographs = Exp{1.124–0.259 Age + 0.010 Age^2^}.

The next analysis investigated whether the rate of chest radiographs (mean number divided by age in years) differed with age. As shown in Figure 1, a negative binomial model fit the data. The deviance/degrees of freedom (df) was 1.029, indicating the chi-square test of goodness-of-fit was non-significant (deviance = 246.016, df = 239, *P* = 0.223). Akaike Information Criterion (AIC) and Bayesian Information Criterion (BIC) for the negative binomial model were smaller than those for the Poisson model: AIC = 1280.94 vs. 1549.00 and BIC = 1294.75 vs. 1559.38. Thus, the negative binomial model fit the data better than the Poisson model. The rate of chest radiographs per year was significantly different for children of different ages (likelihood ratio chi-square test = 56.61, df = 2, *P* = 0.000). This rate was negatively related to the child's age. The younger the child, the higher the rate (model coefficient of age = −0.259, *P* = 0.000; and model coefficient of age^2^ = 0.010, *P* = 0.016), Supplementary Table 4 (Supplementary Material). Gender was not significant when added to the negative binomial model described above (*P* = 0.336). Thus, gender was dropped from the model.

## Discussion

The GINA Report defines *asthma exacerbation* as “an acute or subacute deterioration of symptoms and lung function from the baseline control”^2^. As shown here, this term was used imprudently, as the majority (400 of 643, or 62%) of our admissions with this label had considerable comorbidities and, thus, were unfairly designated as asthma exacerbation. Therefore, we have used the following joint criteria for the study eligibility: (1) Use of systemic corticosteroids for the management of asthma exacerbation and (2) Absence of associated (relevant) clinical conditions that could influence the assessment and management. A misuse of the diagnostic tag “asthma exacerbation” impedes the development and implementation of practical evidence-based rules for these children. In addition, variations linked to regional medical resources (e.g., national health coverage vs. self-paid) play an important role in these admissions. In a recent study that involved several emergency departments, the prevalence of chest radiograph use in children with asthma was about 30% (12). This practice did not significantly change after implementing a quality improvement approach (pathway) (12).

Radiation is a well-known cause of cancer, especially for *high* exposures in early life (13). In our study, young children with asthma received significantly more chest radiographs than older children. Therefore, effort should be taken to alter the current practice and habit of unnecessary use of medical radiation (including computerized tomography scan) on these young children (14, 15). Suggested recommendations toward this goal include: (1) Educating healthcare providers about potential adverse events coupled with diagnostic radiation (e.g., the AAP initiative for “improving the value of care delivered to children with asthma”)^3^ (12), (2) Relying on the medical history and physical examination to attain clinical assessment (16, 17), (3) Regulating radiation equipment to deliver minimum necessary dosing (13), (4) Establishing evidence-based thresholds for the allowed radiation dose as function of age (18), and (5) Advancing ultrasound and magnetic resonance imaging (MRI) technologies to replace the diagnostic radiation procedures.

This study also shows that the significant predictor for using antibiotics was abnormal chest radiograph. Therefore, proper interpretation of chest radiographs is essential to minimize the use of antibiotics. As shown here, only 38 (18%) of the 215 chest radiographs showed focal or multifocal pneumonia justifying antibiotic use. Requesting a chest radiograph and using an antibiotic are highly correlated; and whether the decision to use antibiotics increases the request of chest radiograph, or the reverse, cannot be easily determined. This is in part due to the improper interpretations of radiographic findings of asthma and viral infections. This inference is supported by the subsequent (independent) review of the chest radiographs by pediatric radiologist.

Study limitations include being retrospective, single institution experience, limited number of older children (>5 y), not including infants (<1 y) and young toddlers (1 to 2 y), not being a blinded trial with respect to the chest radiograph assessment, and missing the total radiation dose from all diagnostic procedures. These issues are subjects of future research. Although the presented data may reflect mainly our local practice, we hope these results spike interest for similar studies at other institutions to assess the variation in asthma care.

The critical quality improvement concern is how to motivate care providers to comprehend and implement the GINA guidelines in their management of childhood asthma, including requesting chest radiographs for the exacerbation episodes. Toward this purpose, a short survey has been developed (Supplementary Material) to be completed before and after genuine engagements with GINA-based educational materials. Prospective studies are also needed to assess the outcome of these quality improvement initiatives.

## Conclusions

Evidence-based guidelines are needed to minimize medical radiation, especially in young children. Educating and counseling parents is crucial to achieve this goal. The electronic medical record should alert healthcare providers on the cumulative medical radiation (yellow or red) and should only proceed with a valid reason for the imaging. Evidence-based guidelines are also needed to define the proper assessment and management of children hospitalized for asthma.

## Data Availability Statement

The original contributions presented in the study are included in the article/Supplementary Material, further inquiries can be directed to the corresponding author/s.

## Ethics Statement

The studies involving human participants were reviewed and approved by Tawam Human Research Ethics Committee. Written informed consent from the participants' legal guardian/next of kin was not required to participate in this study in accordance with the national legislation and the institutional requirements.

## Author Contributions

A-KS, EB, and HA: conceptualization. EB, SJ, MM, HMA, OH, GG, and AA: data curation. TZ: statistical analysis. MT: radiographic analysis. EB, A-KS, HA, and MA: clinical data analysis. A-KS and EB: writing—original draft preparation. A-KS, EB, TZ, MT, and HA: writing—review & editing. All authors contributed to the article and approved the submitted version.

## Conflict of Interest

The authors declare that the research was conducted in the absence of any commercial or financial relationships that could be construed as a potential conflict of interest.

## Publisher's Note

All claims expressed in this article are solely those of the authors and do not necessarily represent those of their affiliated organizations, or those of the publisher, the editors and the reviewers. Any product that may be evaluated in this article, or claim that may be made by its manufacturer, is not guaranteed or endorsed by the publisher.

## Footnotes

^1^https://www.fda.gov/radiation-emitting-products/medical-imaging/pediatric-x-ray-imaging. (accessed April 15, (2021).

^2^ https://ginasthma.org/wp-content/uploads/2019/01/2018-GINA.pdf

^3^https://www.aappublications.org/news/2020/05/12/inpatient-asthma-care-pediatrics-5-12-20 (accessed April 6, 2021).
