# Supplementary material for: A Single-Institution Experience in the Use of Chest Radiographs for Hospitalized Children Labeled as Asthma Exacerbation
Source: Front Pediatr. 2021 Aug 19;9:722480. doi: 10.3389/fped.2021.722480 (PMC8416998; doi:10.3389/fped.2021.722480)
Supplement: Supplementary file 1 [file Data_Sheet_1.PDF]

A Single-Institution Experience in the Use of Chest Radiographs for Hospitalized Children Labelled as Asthma Exacerbation

Ela Beyyumi[1,†], Mohamed I. Tawil[2,†], Huda AlDhanhani[1,*], Sara Jameel[1], Manal Mouhssine[1], Hasa M. AlNuaimi[1], Osama Hamdoun[1], Amnah Alabdouli[1], Mohammed T. Alsamri[1], Ghassan A. Ghatasheh[1], Taoufik Zoubeidi[3], Abdul-Kader Souid[4]

[1] Department of Pediatrics, Tawam Hospital, Alain, Abu Dhabi, UAE

[2] Department of Radiology, Sheikh Khalifa Medical City, Abu Dhabi, UAE

[3] Department of Statistics, College of Business and Economics, UAE University, Al Ain, UAE

[4] Department of Pediatrics, College of Medicine & Health Sciences, UAE University, Al Ain, UAE

[†]These authors have contributed equally to this work and share first authorship.

Ela Beyyumi, ORCID: 0000-0001-7077-2338; Ebeyyumi@seha.ae
Mohamed I. Tawil; ORCID: 0000-0003-3605-5495; mtawil@seha.ae
Huda AlDhanhani; hdhanhani@seha.ae
Sara Jameel; sjameel@seha.ae.
Manal Mouhssine; mmouhssine@seha.ae
Hasa M. AlNuaimi; hasnuaimi@seha.ae
Osama Hamdoun; oshamdoun@seha.ae
Amnah Alabdouli; https://orcid.org/0000-0001-5786-3374; asmabdouli@seha.ae
Mohammed T. Alsamri, ORCID: 0000-0003-2475-7967; malsamri@seha.ae
Ghassan A. Ghatasheh; gghatasheh@seha.ae
Taoufik Zoubeidi; taoufikz@uaeu.ac.ae
Abdul-Kader Souid, ORCID: 0000-0002-8562-4757; asouid@uaeu.ac.ae

Correspondences: Huda Aldhanhani (hdhanhani@seha.ae)

Running head: Clinical care of hospitalized children for asthma.

SUPPLEMENTARY MATERIAL

Table 4S (Supplementary Material): Parameter estimates of the rate of chest radiographs as function of age and age^2.

Parameter	B	Std. Error	95% Profile Likelihood Confidence Interval		Hypothesis Test		
			Lower	Upper	Wald Chi-Square	df	*P*
Intercept	1.124	0.1853	0.761	1.490	36.778	1	0.000
Age	-0.259	0.0612	-0.379	-0.138	17.850	1	0.000
Age^2	0.010	0.0041	0.002	0.018	5.792	1	0.016
Negative binomial	0.418	0.0546	0.322	0.538			

Dependent variable: Number of chest radiographs since birth. *Model*: (Intercept), Age, Age^2, offset = natural logarithm (ln) of age.

ASTHMA SURVEY

Date: ; Student – Resident - Post-residency (please circle one)

PART I

Please circle (or check) all that apply to the "*care of a child with asthma exacerbation*":

Chest radiograph (single view)
Chest radiograph (two views)
Antibiotics
Laboratory investigation
Blood culture
Venous (capillary) blood gases
Nasopharyngeal swab for pathogen detection
Pulse oximeter
Oxygen
Intravenous fluid
Admission
Close observation
Review compliance with the asthma plan
Contact the primary physician
Review clinical data of prior admissions
Review radiographs of prior admissions
Review laboratory results of prior admissions
Review growth and development
Undress and inspect the chest
Inspect for finger clubbing
Obtain family history of pulmonary disease
Consult the Global Initiative for Asthma (GINA) guidelines

PART II

1. Are you aware of the Global Initiative for Asthma (GINA)?
   Yes
   No
2. Do you know website links to GINA?
   Yes
   No
3. In the past 12 months, have you accessed any GINA website?
   Yes
   No
4. Have you ever used GINA to guide your management of asthma?
   Yes
   No
5. In the past 12 months, have you used GINA for the management of asthma?
   Yes
   No
6. What would you suggest to improve the accessibility of GINA? (please type in your response)

7. Do you use alternative sources other than GINA to guide your management of asthma?
   Yes (please specify)
   No
